# Electronic Cigarette Liquid Increases Inflammation and Virus Infection in Primary Human Airway Epithelial Cells

**DOI:** 10.1371/journal.pone.0108342

**Published:** 2014-09-22

**Authors:** Qun Wu, Di Jiang, Maisha Minor, Hong Wei Chu

**Affiliations:** Department of Medicine, National Jewish Health, Denver, Colorado, United States of America; Louisiana State University, United States of America

## Abstract

**Background/Objective:**

The use of electronic cigarettes (e-cigarettes) is rapidly increasing in the United States, especially among young people since e-cigarettes have been perceived as a safer alternative to conventional tobacco cigarettes. However, the scientific evidence regarding the human health effects of e-cigarettes on the lung is extremely limited. The major goal of our current study is to determine if e-cigarette use alters human young subject airway epithelial functions such as inflammatory response and innate immune defense against respiratory viral (i.e., human rhinovirus, HRV) infection.

**Methodology/Main Results:**

We examined the effects of e-cigarette liquid (e-liquid) on pro-inflammatory cytokine (e.g., IL-6) production, HRV infection and host defense molecules (e.g., short palate, lung, and nasal epithelium clone 1, SPLUNC1) in primary human airway epithelial cells from young healthy non-smokers. Additionally, we examined the role of SPLUNC1 in lung defense against HRV infection using a SPLUNC1 knockout mouse model. We found that nicotine-free e-liquid promoted IL-6 production and HRV infection. Addition of nicotine into e-liquid further amplified the effects of nicotine-free e-liquid. Moreover, SPLUNC1 deficiency in mice significantly increased lung HRV loads. E-liquid inhibited SPLUNC1 expression in primary human airway epithelial cells. These findings strongly suggest the deleterious health effects of e-cigarettes in the airways of young people. Our data will guide future studies to evaluate the impact of e-cigarettes on lung health in human populations, and help inform the public about potential health risks of e-cigarettes.

## Introduction

Electronic cigarettes (e-cigarettes) are battery-operated devices that heat up e-cigarette liquid (e-liquid) without or with nicotine and turn it into an inhalable vapor. Perceived as a safer alternative to conventional tobacco cigarettes, the use of e-cigarettes has increased rapidly in the United States (U.S.). Despite banning e-cigarette sales to minors in some U.S. states, e-cigarettes can be purchased in bordering states or via the Internet [1]. As a result, a notable proportion of adolescents and young adults who had never smoked tobacco cigarettes have used e-cigarettes. About 1.78 million U.S. youth had ever used e-cigarettes as of 2012 [2]. While e-cigarette manufacturers claim that their products are harmless, adverse respiratory effects (e.g., cough, wheezing and pneumonia) have been reported in social media from e-cigarette users [3]. However, the scientific evidence regarding the human health effects of e-cigarettes on the lung is extremely limited.

Airway epithelial cell is the primary target for any inhaled environmental agents including tobacco cigarette smoke [4]–[6]. Tobacco cigarette smoke is well known to cause a wide range of respiratory problems such as airway inflammation and increased incidence and severity of respiratory viral infections [7]–[13]. Human rhinovirus (HRV) is the most common pathogen of acute infections in upper respiratory tract (e.g., common cold) and can provoke acute exacerbations of lower airway diseases such as asthma and chronic obstructive pulmonary disease (COPD) [14]. Pro-inflammatory cytokine IL-6 is correlated to the levels of tobacco cigarette smoke exposure and contributes to acute lung inflammation in tobacco cigarette smokers [15], [16]. More importantly, IL-6 plays an important role in the progression of COPD severity. For example, increased sputum IL-6 levels have been found in COPD smokers, particularly during virus-induced exacerbations [17]–[19].

To date, whether e-cigarettes share some of the adverse effects of tobacco cigarettes remains unclear. Studies by the Food and Drug Administration (FDA) show that e-cigarette vapor contains some of the same toxic chemicals as tobacco cigarettes [20]. E-cigarette vapor produces small particles that are similar to those contained in tobacco cigarette smoke and able to deposit on airway epithelium [21]. A short exposure to propylene glycol, the primary ingredient in the majority of e-liquid and e-cigarette cartridges, may cause acute upper airway irritation in human non-smokers [22]. E-cigarettes have also been shown to induce carcinogenicity-related gene expression and transformation of immortalized human bronchial epithelial cells in a similar way to tobacco cigarettes [23]. However, there is essentially no data describing the effects of e-cigarettes on human airway epithelial functions such as inflammatory response and innate immune defense against respiratory viral (i.e., HRV) infections.

In the present study, we hypothesized that e-cigarettes have detrimental effects on human airway epithelial functions. We examined the effects of e-liquid on the production of pro-inflammatory cytokine IL-6, HRV infection and the expression of host defense molecules (e.g., short palate, lung, and nasal epithelium clone 1, SPLUNC1) in primary human airway epithelial cells from young healthy non-smokers. Additionally, we confirmed the beneficial role of SPLUNC1 in lung defense against HRV infection using a SPLUNC1 knockout mouse model.

## Materials and Methods

### E-liquid exposure in normal human tracheobronchial epithelial (hTBE) cells

Normal hTBE cells were isolated from the tracheas and bronchi of de-identified organ donors (8–10 years old) whose lungs were not suitable for transplantation and donated for medical research as described previously [24]. We obtained the donor lungs through the International Institute for the Advancement of Medicine (Edison, NJ) and the National Disease Research Interchange (Philadelphia, PA). Our collection of hTBE cells was approved by the Institutional Review Board (IRB) of National Jewish Health. Briefly, tracheas and bronchi were cut into small pieces and digested with ice-cold DMEM supplemented with 0.2% protease solution (Sigma-Aldrich, St. Louis, MO) and 1× pen/strep/amphotericin (HyClone, Thermo Scientific, Logan, UT) overnight at 4°C. The released cells were cultured in collagen-coated 60 mm tissue culture dishes containing bronchial epithelial cell growth medium (BEGM) with supplements (Lonza, Walkersville, MD) at 37°C, 5% CO_2_. Cells at passage 2 were seeded into 24-well cell culture plates at 1×10^5^ cells/well in BEGM with supplements at 37°C, 5% CO_2_. At 70−80% confluence, cells were treated with medium, tobacco-flavored e-liquid at various concentrations (0, 0.01, 0.1, 0.3% v/v) without nicotine or with 18 mg/ml of nicotine (InnoVapor LLC., Boise, ID) for 24 and 48 h. The final nicotine concentrations were within the serum nicotine range of e-cigarette users [25]–[29]. At the end of culture, cell culture supernatants were collected to assess cell toxicity by measuring lactate dehydrogenase (LDH) levels and IL-6 protein levels by ELISA.

### HRV preparation

HRV-16 and HRV-1B (American Type Culture Collection, Manassas, VA) were propagated in H1-Hela cells (CRL-1958, ATCC), purified and titrated as described previously [30].

### HRV-16 infection in e-liquid-exposed normal hTBE cells

To examine the effects of e-liquid exposure on inflammation and HRV-16 infection, hTBE cells from young healthy non-smokers at passage 2 were seeded into 24-well cell culture plates at 1×10^5^ cells/well in BEGM with supplements at 37°C, 5% CO_2_. At 70−80% confluence, cells were pre-treated with medium, tobacco-flavored e-liquid at an optimized concentration (0.3% v/v) without nicotine or with 18 mg/ml of nicotine for 24 h. Then, cells were infected with HRV-16 at 10^4^ TCID_50_/well or PBS (control) for additional 6 and 24 h. At the end of culture, cell culture supernatants were collected to measure IL-6 protein levels by ELISA. Cells were processed to examine HRV RNA and human SPLUNC1 mRNA by quantitative real-time RT-PCR.

### HRV-1B infection in mice

SPLUNC1 knockout (KO) and littermate control wild-type (WT) mice on the C57BL/6 background were generated as we previously reported [31]. All mice were bred and housed in biological resource center under pathogen-free conditions, and tested to establish that they were virus and *M. pulmonis* free. All the animal procedures were approved by the Institutional Animal Care and Use Committee (IACUC) at National Jewish Health.

SPLUNC1 KO and control mice (8–12 weeks old) were anesthetized by intra-peritoneal (i.p.) injection of ketamine (80 mg/kg) and xylazine (10 mg/kg), and intranasally inoculated with HRV-1B at 5×10^6^ pfu/mouse as HRV-1B is the only HRV that can directly infect mouse lungs [32]. After 24 h, the left lung was homogenized for examining HRV RNA levels by quantitative real-time RT-PCR.

### Lactate dehydrogenase (LDH) assay

To quantitate the cytotoxic effects of e-liquid exposure, cell culture supernatants were subjected to measure LDH levels using a cytotoxicity detection kit (Roche Diagnostics, Indianapolis, IN) according to the manufacturer’s instruction. OD value at 450 nm was measured using a microplate reader. Data were expressed as the percentage cytotoxicity of cells with various treatments as compared to medium alone.

### ELISA

IL-6 protein levels in cell culture supernatants were determined by using the Ready-Set-Go Human IL-6 ELISA (eBioscience Inc., San Diego, CA) as per the manufacturer’s instruction.

### Quantitative real-time RT-PCR

Taqman quantitative real-time RT-PCR was used to detect HRV RNA [33] and human SPLUNC1 mRNA [34] as described previously. The specific primers and probes were: HRV (forward: 5′-CCTCCGGCCCCTGAAT-3′; reverse: 5′-GGTCCCATCCCGCAATT-3′, probe: 5′-CTAACCTTAAACCTGCAGCCA-3′) and human SPLUNC1 (forward primer, 5′-GGGCCTGTTGGGCATTCT-3′; reverse primer, 5′-CCTCCTCCAGGCTTCAGGAT-3′; probe, 5′-AAACCTTCCGCTCCTGGA-3′). Housekeeping gene GAPDH in human samples or 18S rRNA in mouse samples was evaluated as an internal positive control. The comparative cycle of threshold (ΔΔCt) method was used to demonstrate the relative levels of target genes.

### Statistical analysis

Data are presented as means ± SEM. One-way analysis of variance (ANOVA) was used for multiple comparisons, and a Tukey’s post hoc test was applied where appropriate. Student’s *t* test was used when only two groups were compared. A *p* value<0.05 was considered significant.

## Results

### E-liquid induces IL-6 production in primary human airway epithelial cells

**Figure 1:**
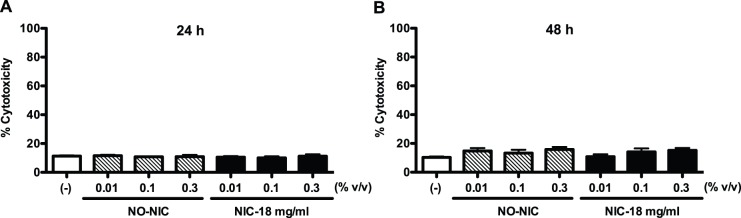
The physiological doses of e-liquid do not decrease primary human airway epithelial cell viability. Normal human tracheobronchial epithelial cells from young healthy non-smokers were treated with medium (control), e-liquid at various concentrations without nicotine (NO-NIC) or with 18 mg/ml of nicotine (NIC-18 mg/ml) for 24 h (A) and 48 h (B). Cell toxicity was assessed by measuring lactate dehydrogenase (LDH) levels in cell culture supernatants. Data (n = 4) are presented as mean ± SEM.

LDH release is an indicator of membrane integrity and viability of cells including airway epithelial cells [35]–[37]. We measured LDH levels in cell culture supernatants under various treatment conditions. Within the physiological nicotine range, e-liquid exposure did not cause noticeable cytotoxicity at both 24 and 48 h (Figure 1).

**Figure 2:**
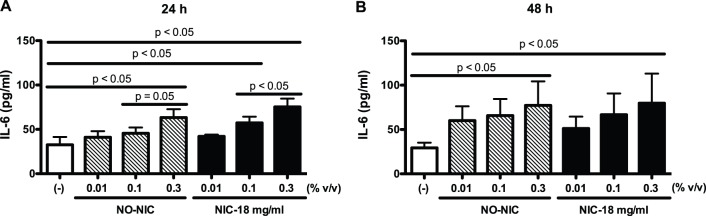
E-liquid induces IL-6 production in primary human airway epithelial cells. Normal human tracheobronchial epithelial cells from young healthy non-smokers were exposed to medium (control), e-liquid at various concentrations without nicotine (NO-NIC) or with 18 mg/ml of nicotine (NIC-18 mg/ml) for 24 h (A) and 48 h (B). IL-6 protein levels in cell culture supernatants were measured by ELISA. Data (n = 4) are presented as means ± SEM.

To determine if e-cigarette use induces inflammation, hTBE cells from young healthy non-smokers were exposed to e-liquid at various concentrations (0, 0.01, 0.1, 0.3% v/v) without nicotine or with 18 mg/ml of nicotine to examine IL-6 production for 24 and 48 h. Exposure to e-liquid without nicotine increased IL-6 protein levels in a dose-dependent manner at both 24 and 48 h (Figure 2). Addition of nicotine to e-liquid only marginally enhanced the IL-6 levels. As e-liquid treatment (without and with nicotine) at 0.3% v/v concentration induced the highest production of IL-6 as early as 24 h, incubation with e-liquid at 0.3% v/v for 24 h was chosen as the optimized condition for the subsequent experiments in this study.

### E-liquid promotes HRV infection in primary human airway epithelial cells

To date, there is no data describing the effects of e-cigarette use on respiratory viral (i.e., HRV) infections in human primary airway epithelial cells. We infected hTBE cells from young healthy non-smokers with HRV-16, a major group HRV commonly seen in human subjects, in the absence or presence of e-liquid pre-treatment to examine HRV load and IL-6 production for 6 and 24 h. The dose of 10^4^ TCID_50_/well for HRV-16 was chosen based on our previous publication in human lung epithelial cell cultures [30]. The 6 and 24 h time points were chosen according to our preliminary time-course (6, 24 and 48 h) optimization experiments where cells were infected with HRV-16 at the dose of 10^4^ TCID_50_/well. We found that HRV-16 levels were increased at 6 h, and maintained at 24 h, but not at 48 h.

**Figure 3:**
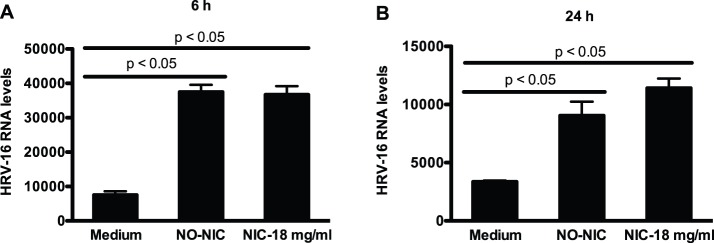
E-liquid promotes human rhinovirus (HRV) infection in primary human airway epithelial cells. Normal human tracheobronchial epithelial cells from young healthy non-smokers were pre-exposed to medium (control), e-liquid at an optimized concentration (0.3% v/v) without nicotine (NO-NIC) or with 18 mg/ml of nicotine (NIC-18 mg/ml) for 24 h and then infected with HRV-16 at 10^4^ TCID_50_/well or PBS (control) for 6 h (A) and 24 h (B). Viral RNA levels were measured by quantitative real-time RT-PCR. Data (n = 5) are presented as means ± SEM.

At the time of HRV infection, all the cells reached the similar confluence (about 90−100%). No significant difference in cell growth (density) was observed during HRV infection period. As shown in Figure 3A and 3B, cells exposed to tobacco-flavored e-liquid (without or with nicotine) had higher levels of HRV load than unexposed cells at both 6 and 24 h. Compared with nicotine-free e-liquid, addition of nicotine into e-liquid either did not alter (6 h) or slightly (p>0.05) increased (24 h) HRV load.

**Figure 4:**
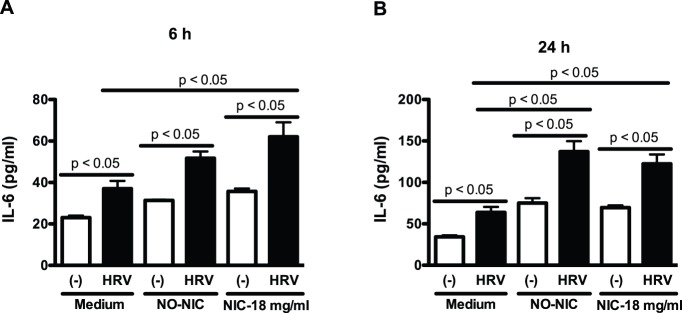
E-liquid enhances human rhinovirus-induced IL-6 production in primary human airway epithelial cells. Normal human tracheobronchial epithelial cells from young healthy non-smokers were pre-exposed to medium (control), e-liquid at an optimized concentration (0.3% v/v) without nicotine (NO-NIC) or with 18 mg/ml of nicotine (NIC-18 mg/ml) for 24 h and then infected with HRV-16 at 10^4^ TCID_50_/well or PBS (control) for 6 h (A) and 24 h (B). IL-6 protein levels in cell culture supernatants were measured by ELISA. Data (n = 5) are presented as means ± SEM.

Moreover, HRV infection significantly increased IL-6 production at both 6 and 24 h in cells that were pre-exposed to medium alone or e-liquid without and with nicotine. At 6 h, only e-liquid with nicotine significantly enhanced HRV-induced IL-6 production as compared to medium-treated and HRV-infected cells (Figure 4A). However, at 24 h, both e-liquid without nicotine and with nicotine similarly augmented HRV-induced IL-6 production (Figure 4B).

### E-liquid inhibits the expression of SPLUNC1, a host defense molecule against HRV infection

**Figure 5:**
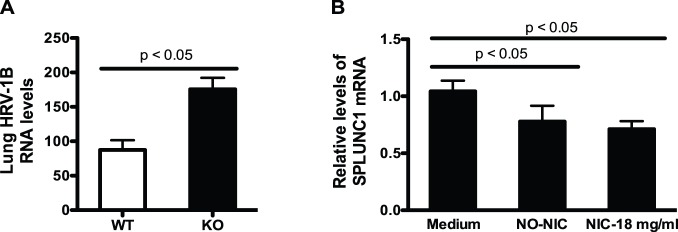
E-liquid inhibits SPLUNC1, a host defense molecule against human rhinovirus infection, in primary human airway epithelial cells. (A) SPLUNC1 knockout (KO, n = 5) mice and littermate controls (WT, n = 5) were intranasally inoculated with HRV-1B at 5×10^6^ pfu/mouse for 24 h to examine lung HRV RNA levels by quantitative real-time RT-PCR. Data are presented as means ± SEM. (B) Normal human tracheobronchial epithelial cells from young healthy non-smokers were pre-exposed to medium (control), e-liquid at an optimized concentration (0.3% v/v) without nicotine (NO-NIC) or with 18 mg/ml of nicotine (NIC-18 mg/ml) for 24 h and then infected with HRV-16 at 10^4^ TCID_50_/well or PBS (control) for 6 h. SPLUNC1 mRNA levels were measured by quantitative real-time RT-PCR. Data (n = 5) are presented as means ± SEM.

SPLUNC1 has been recognized as an important antimicrobial protein in airways against various bacterial infections [31], [38]–[42]. Although decreased SPLUNC1 has been reported in the upper airways of respiratory syncytial virus (RSV)-infected infants [43], its role in lung defense against HRV infection remains unclear. To unequivocally determine a role of SPLUNC1 against lung HRV infection, SPLUNC1 KO mice and littermate controls were intranasally inoculated with HRV-1B for 24 h. As shown in Figure 5A, SPLUNC1 KO mice had significantly higher HRV loads in lung tissue than the control mice. Having shown a beneficial role of SPLUNC1 in lung defense against HRV infection, we then examined the impact of e-liquid on SPLUNC1 mRNA expression in hTBE cells from young healthy non-smokers at 6 h post HRV-16 infection. Compared with medium controls, SPLUNC1 mRNA expression was significantly reduced by e-liquid without nicotine and with nicotine (Figure 5B). This finding is similar to the previous data that SPLUNC1 is decreased by tobacco cigarette smoke exposure [44], [45].

## Discussion

This is the first study to demonstrate the adverse effects of e-cigarettes on primary airway epithelial functions from young people. Our data suggest that even nicotine-free e-liquid promotes pro-inflammatory response and HRV infection. Moreover, both e-liquid without nicotine and with nicotine inhibits lung innate immunity (e.g., SPLUNC1) that is involved in lung defense against HRV infection.

The research regarding the detrimental health effects of e-cigarettes is still in its infancy. Several recent studies have compared the cytotoxic effects of e-cigarettes vs. conventional tobacco cigarettes. Their results demonstrate that exposure to e-cigarette vapor with nicotine results in far less cytotoxic effects (e.g., cell viability, cell ultrastructure and pro-inflammatory cytokines) than exposure to tobacco cigarette smoke in human lung epithelial cell line, human keratinocyte cell line, rat cardiomyoblast cell line and murine fibroblast cell line [46]–[48]. For the first time, we have investigated the adverse effects of e-cigarettes without nicotine and with nicotine on airway epithelial functions by using primary human airway epithelial cells. Our results have clearly demonstrated that primary human airway epithelial cells expressed higher IL-6 protein levels upon exposure to e-liquid even without nicotine. Interestingly, addition of nicotine into e-liquid slightly enhanced IL-6 production. We also found that the treatments of e-liquid without nicotine and with nicotine similarly enhanced production of IL-8, another important pro-inflammatory cytokine induced by tobacco smoke exposure and/or HRV infection [49], [50]. As pure nicotine has anti-inflammatory effects in human bronchial epithelial cell cultures [6], [51], this mild pro-inflammatory effect of nicotine in e-liquid might be related to other factors such as endotoxin contamination during the e-liquid manufacturing. Future studies using polymyxin B to remove any potential endotoxin in e-liquid with nicotine would help test this possibility.

Tobacco cigarette smoke-mediated inflammatory responses in the airways are important in the pathogenesis of smoking-related lung diseases. IL-6 is one of the important pro-inflammatory cytokines in COPD smokers, particularly during virus-induced exacerbations [17]–[19]. Among various respiratory viruses, HRV is the most common trigger for exacerbations in COPD smokers [52]–[55]. To date, there is no data describing the effects of e-cigarettes on respiratory viral (i.e., HRV) infections in human airways. In the current study, we have disclosed that e-liquid without nicotine and with nicotine both significantly increased HRV load in primary human airway epithelial cells, which is accompanied with enhanced production of HRV-induced IL-6. This suggests that e-cigarette use may promote respiratory viral infections and exaggerate airway inflammation in a similar manner to tobacco cigarette smoking.

How e-cigarettes promote airway epithelial HRV infection remains unclear. In the current study, for the first time, we have demonstrated a significant increase of lung HRV load in SPLUNC1 KO mice compared with wild-type mice. Moreover, we found that e-liquid without nicotine and with nicotine both inhibit SPLUNC1 mRNA expression in cultured primary human airway epithelial cells. Taken together, our findings highlight a beneficial role of SPLUNC1 in host defense against HRV infection in airways, and a potential novel mechanism by which e-cigarettes promote lung HRV infection.

There are several limitations to our present study. First, many e-cigarette ingredients (e.g., propylene glycol) have been approved by the FDA for human consumption as liquid, but their safety as a vapor for inhalation has not been studied. In our recent pilot study, we have exposed well-differentiated young hTBE cells at the air-liquid interface (ALI) culture to tobacco-flavored e-cigarette vapor by using a cell exposure chamber as reported in our previous tobacco cigarette smoke research [56]–[58]. We found that a 10-min exposure of e-cigarette vapor without nicotine and in particular with nicotine (18 mg/cartridge) significantly increased IL-6 protein levels after 24 h. Future research will be pursued to examine the effects of e-cigarette vapor with various flavors and nicotine strengths on epithelial functions in the absence or presence of HRV infection in well-differentiated primary human airway epithelial cells. Second, the current study focused on the acute exposure effect of e-liquid on airway epithelial functions. Repeated exposures to e-liquid or e-cigarette vapor in the absence or presence of HRV infection *in* *vitro* and *in* *vivo* will be pursued to study the chronic effects of e-cigarette use on airway epithelial functions. Third, as the focus of our current study is to determine the adverse effects of e-cigarettes on airway epithelial functions, we did not examine the signaling pathways underlying IL-6 up-regulation by e-liquid treatment. Nonetheless, future studies are warranted to reveal the molecular mechanisms of IL-6 regulation by e-cigarettes.

In summary, our current study has provided strong data suggesting the deleterious health effects of e-cigarettes on the lung, with a particular focus on airway epithelial inflammation and innate immunity in young people. Our research findings will guide future studies to evaluate the impact of e-cigarettes on lung health in human populations, in particular the youth, which will ultimately help inform the public about potential health risks of e-cigarettes.
